# Elevation of Serum APE1/Ref-1 in Experimental Murine Myocarditis

**DOI:** 10.3390/ijms18122664

**Published:** 2017-12-08

**Authors:** Seon-Ah Jin, Byung-Kwan Lim, Hee Jung Seo, Sun Kyeong Kim, Kye Taek Ahn, Byeong Hwa Jeon, Jin-Ok Jeong

**Affiliations:** 1 Division of Cardiology, Department of Internal Medicine, Chungnam National University Hospital, Chungnam National University School of Medicine, 282 Munhwa-ro, Jung-gu, Daejeon 35015, Korea; drjsa@cnuh.co.kr (S.-A.J.); jjeong03@nate.com (H.J.S.); sunajin@hanmail.net (S.K.K.); kapula@cnuh.co.kr (K.T.A.); 2 Department of Biomedical Science, Jungwon University, Goesan-gun 28024, Korea; bklim@jwu.ac.kr; 3 Department of Physiology, Chungnam National University Hospital, Chungnam National University School of Medicine, 266 Munhwa-ro, Jung-gu, Daejeon 35015, Korea; bhjeon@cnu.ac.kr

**Keywords:** Ape1 protein, mouse, apurinic/apyrimidinic endonuclease 1/redox effector factor-1(APE1/Ref-1), biomarkers, heart failure, myocarditis, Ref-1 protein, mouse, coxsackievirus

## Abstract

Myocarditis is an inflammatory disease of the myocardium that causes cardiogenic shock and death. However, endomyocardial biopsy that is, the gold standard for a diagnosis is limited. Apurinic/apyrimidinic endonuclease 1/redox effector factor-1 (APE1/Ref-1) is a multifunctional protein, which is involved in DNA-based excision repair pathway, and in redox signaling, its changes are observed in various cardiovascular diseases including hypertension and coronary artery disease. We analyzed serum APE1/Ref-1 in experimental murine myocarditis. To induce myocarditis, coxsackievirus B3 was injected intraperitoneally to BALB/c mice. The serum APE1/Ref-1, N-terminal pro-B-type natriuretic peptide (NT-proBNP) and troponin I were measured. The histology and virus titers measurements were performed. The troponin I and inflammation were significantly elevated at day 3, peaked to day 7 and decreased at day 10. The NT-proBNP and virus titers were significantly peaked at day 3, and dropped at day 7 and 10. The serum APE1/Ref-1 was gradually raised and its elevation is still maintained until a later time, namely day 10. Also, its level was positively correlated with myocardial inflammation, reflecting severity of myocardial injury. We suggest that serum APE1/Ref-1 can be used to assess for myocardial injury in viral myocarditis without endomyocardial biopsy.

## 1. Introduction

Myocarditis is an inflammatory disease of the myocardium, which can be produced with a variety of different causes. Clinical features in myocarditis are polymorphic, ranging from no symptom to cardiogenic shock and death. Most often, myocarditis results in chronic heart failure [1]. However, there are great difficulties in establishing a diagnosis and evaluating prognosis of myocarditis. Endomyocardial biopsy (EMB) is a diagnostic gold standard but, it is an invasive test and its sensitivity is very low because of variability in interpretation and sampling error [2,3]. The diagnosis based on clinical presentation alone is unreliable.

Cardiac biomarkers are used to establish a diagnosis and to stratify risk in various diseases including heart failure, ischemic heart disease and myocarditis [4]. Elevation of troponin T, I and high-sensitivity troponin are observed in some patients of myocarditis because of cardiomyocyte injury [5]. N-terminal pro-B-type natriuretic peptide (NT-proBNP) can be elevated as a result of cardiomyocyte strain in myocarditis. However, these markers are not widely useful in myocarditis. Because normal values in these markers can’t rule out myocarditis and their elevated values can’t reflect the degree of myocardial inflammation [6]. Also, the elevation of inflammatory markers and cytokines such as erythrocyte sedimentation rate, C-reactive protein, interleukin-1α, tumor necrosis factor and interferon gamma are often reported in myocarditis. But, they can’t confirm the diagnosis and help to predict prognosis [7,8]. Other serum immunological biomarkers including complement and anti-heart antibodies have not been validated for accurate screen in myocarditis [9,10].

Apurinic/apyrimidinic endonuclease 1/redox effector factor-1 (APE1/Ref-1) is a multifunctional protein that is mainly located in nucleus. APE1/Ref-1 is involved in repair pathway to DNA damage and redox regulation to oxidative stress. Altered expression, activity and localization of APE1/Ref-1 have been reported in various diseases such as hypertension, carotid atherosclerotic plaque and coronary artery disease [11,12,13,14,15,16]. Also, APE1/Ref-1 was known that it exhibits anti-inflammatory functions in the vascular endothelium of rats by inhibiting balloon-injury-induced neointimal formation [13]. However, there are no studies about relationship between serum APE1/Ref-1 and myocarditis.

The aim of this study was to examine the possible role of serum APE1/Ref-1 for the diagnosis and evaluation of myocardial inflammation severity in murine acute viral myocarditis models.

## 2. Results

### 2.1. Changes of Serum Troponin I, NT-proBNP and APE1/Ref-1 over Time in Mice with Acute Viral Myocarditis

**Figure 1:**
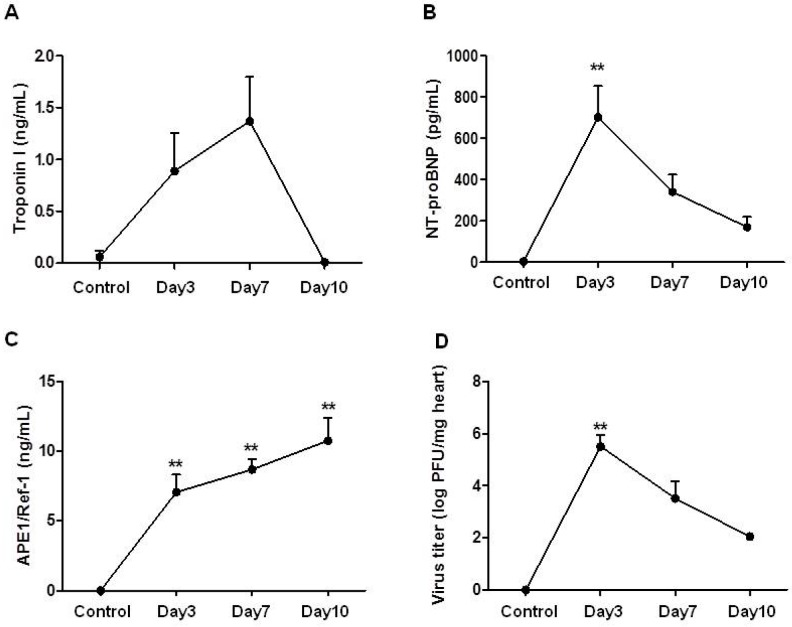
Changes of serum troponin I, NT-proBNP, Apurinic/apyrimidinic endonuclease 1 (APE1)/Ref-1 and heart virus titers over time in mice with acute viral myocarditis. CVB3 was injected intraperitoneally to mice. Blood and hearts were collected after 3, 7 and 10 days of infection. Serum troponin I (**A**) NT-proBNP (**B**) and APE1/Ref-1 (**C**) were increased after the infection and showed respectively different peak time at day 7, 3 and 10 post-infection. In the change of heart virus titer (**D**) maximum virus titers were found after 3 days of the infection. Since then, they had been decreased. In (**A**–**D**), data are presented as mean ± standard error of the mean (SEM) and analyzed by one-way ANOVA, post hoc Dunnett test (*n* = 5 in control; *n* = 10 in day 3; *n* = 10 in day 7; *n* = 7 in day 10). ** *p* < 0.01 vs. control.

The values of serum APE1/Ref-1 showed different pattern from other known cardiac biomarkers (Figure 1). The serum troponin I in mice with acute myocarditis was increased at day 3 (0.06 ± 0.06 ng/mL in control group vs. 0.89 ± 0.37 ng/mL at day 3), reached its peak at day 7 (1.37 ± 0.43 ng/mL), and then normalized at day 10 (0.01 ± 0.0.1 ng/mL) after the infection (Figure 1A). Meanwhile, the serum NT-proBNP was found to peaked at day 3 (4.05 ± 4.05 pg/mL in control group vs. 701.71 ± 151.38 pg/mL at day 3, *p* < 0.01), and this increase was maintained after 10 days of infection (341.28 ± 83.68 pg/mL at day 7 and 170.25 ± 49.73 pg/mL at day 10) (Figure 1B). The serum APE1/Ref-1 was significantly elevated at day 3 (7.05 ± 1.25 ng/mL) and gradually persisted up to day 10 (8.68 ± 0.75 ng/mL at day 7 and 10.74 ± 1.64 ng/mL at day 10, Figure 1C). Namely, the known cardiac biomarkers showed early peak level at day 3 or 7, but the APE1/Ref-1 was increased until the late time point. It suggests that serum APE1/Ref-1 level may be the great indicator at the late time point for viral myocarditis.

### 2.2. Changes of Heart Virus Titers over Time in Mice with Acute Viral Myocarditis

It was well known that the expression of the proinflammatory cytokines in strong association with increasing virus titers at day 3 post-infection. Also, it was reported that inflammatory cytokine expression was decreased after the virus titer declined. To investigate correlation of cardiac marker and virus titer, we measured CVB3 virus titer in the heart at the various time points after infection. The viable CVB3 virus titers in the hearts were shown to be peaked at day 3 post-infection (5.52 ± 0.44 log PFU/mg of heart), and they were decreased gradually (3.52 ± 0.65 log PFU/mg of heart at day 7 and 2.05 ± 0.07 log PFU/mg of heart at day 10). The expression of CVB3 titers at day 3 post-infection was correlated with cardiac markers.

### 2.3. Changes of Histopathological Findings over Time in Mice with Acute Viral Myocarditis

**Figure 2:**
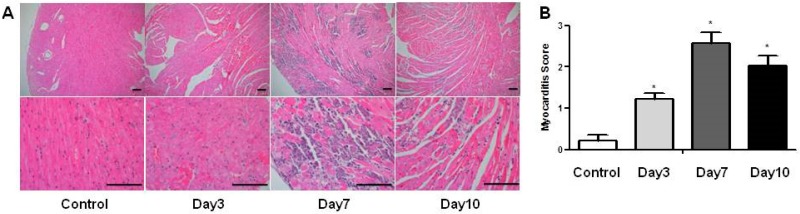
Histopathologic findings of the hearts over time in mice with acute viral myocarditis. (**A**) Changes in histology of heart over time after CVB3 infection (hematoxylin and eosin stain). Scale bar of panel, 100 µm; (**B**) Semiquantitative analysis by the degree of myocardial inflammation. Infiltration of inflammatory cells, myocardial necrosis, fibrosis and mineralization was shown at day 7 after the CVB3 infection. In B, data are presented as mean ± SEM and analyzed by one-way ANOVA, post hoc Dunnett test (*n* = 5 in control; *n* = 10 in day 3; *n* = 10 in day 7; *n* = 7 in day 10). * *p* < 0.05 vs. control group.

Infiltration of inflammatory cells was detected from day 3, peaked at day 7, and declined at day 10 after the infection. This was confirmed by semiquantitative analysis (myocarditis score; 0.22 ± 0.13 in control group vs. 1.23 ± 0.14 at day 3 vs. 2.57 ± 0.26 at day 7 vs. 2.03 ± 0.25 at day 10). At seven days after infection, myocardial necrosis, fibrosis, vacuolization and mineralization with the highest myocarditis score was shown by inflammatory responses (Figure 2). Myocardial inflammation lasted even longer after the virus titer declined at day 3.

### 2.4. Relationship of Serum Troponin I, NT-proBNP, APE1/Ref-1, Viral Titers and Myocardial Inflammation

**Figure 3:**
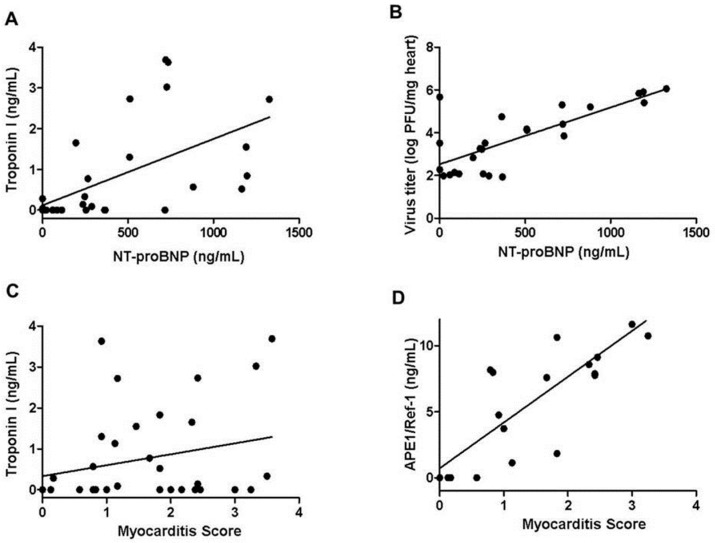
Relationship of serum troponin I, NT-proBNP, APE1/Ref-1, virus titers and myocardial inflammation. (**A**) Serum troponin I was positively correlated with serum NT-proBNP (*r* = 0.586, *p* < 0.01); (**B**) Serum NT-proBNP showed positive association with virus titers (*r* = 0.764, *p* < 0.01); (**C**,**D**) Both Serum troponin I and APE1/Ref-1 indicated positive correlations with the degree of inflammation, myocarditis score. Especially, serum APE1/Ref-1 demonstrated a stronger association (*r* = 0.352, *p* < 0.05 in serum troponin I; *r* = 0.750, *p* < 0.01 in serum APE1/Ref-1). (*n* = 5 in control; *n* = 10 in day 3; *n* = 10 in day 7; *n* = 7 in day 10).

The serum troponin I was correlated with the serum NT-proBNP (*r* = 0.586, *p* < 0.01, Figure 3A). The serum NT-proBNP had positive association with the virus titers of the CVB3 infected hearts (*r* = 0.764, *p* < 0.01, Figure 3B). Both serum troponin I and APE1/Ref-1 were positively correlated with the degree of inflammations in the CVB3 infected hearts, but Pearson’s *r* in serum troponin I was ≤0.40, indicating a weak association (*r* = 0.352, *p* < 0.05, Figure 3C). On the other hand, the serum APE1/Ref-1 showed a strong association with high Pearson’s *r* value (*r* = 0.750, *p* < 0.01, Figure 3D).

## 3. Discussion

In this report, we examined serum APE1/Ref-1 in mice with acute viral myocarditis and evaluated the possible role of it for diagnosing and stratifying myocardial injury, for the first time. We demonstrated that serum APE1/Ref-1 is increased in myocarditis and an elevation of it is maintained till later time when viable virus titers, troponin I and NT-proBNP are decreased or normalized. We also showed that the level of serum APE1/Ref-1 is strongly correlated with myocardial inflammation, reflecting the degrees of myocardial injury.

APE1/Ref-1 is a multifunctional protein that plays an important role in the cellular response to DNA damage and redox regulation against oxidative stress [17]. There are various conditions which show elevated levels of APE1/Ref-1 such as inflammatory status, carotid atherosclerosis, hypertension and coronary artery diseases [12,15,18,19,20]. Jeon et al. reported that heterozygous APE1/Ref-1 mice show impaired endothelium-dependent vasorelaxation, reduced vascular NO levels, and lead to be hypertensive [11]. Martinet et al. described that APE1/Ref-1 is elevated in human carotid atherosclerotic plaques [21]. Although APE1/Ref-1 is predominantly localized to the nucleus, its subcellular localization and extracellularly secretion were reported recently [17,22]. APE1/Ref-1 was secreted into the blood in response to lipopolysaccharides, and Park et al. suggested that plasma APE1/Ref-1 can be used as a serological biomarker for endotoxemia [23]. APE1/Ref-1 showed higher levels in serum from patients with coronary artery disease than from control patients [12]. Although the exact mechanism for translocated or secreted APE1/Ref-1 is not completely understood, a few studies suggested insights into its biological functions. Altered subcellular translocation of APE1/Ref-1 in activated astrocytes regulated anti-inflammatory events [24] and recombinant APE1/Ref-1 showed anti-inflammatory activity by suppressing pro-inflammatory molecules in endothelial cells [23]. Because APE1/Ref-1 is increased and activated under the conditions like reactive oxygen species, ischemia/reperfusion injury, hypoxia or inflammation, its elevation in myocarditis may be expected. Our data confirmed the elevation of APE1/Ref-1 and strong association of it with myocarditis score in mice with acute viral myocarditis. However, serum APE1/Ref-1 is not a just simple inflammation marker. It had no correlation with high sensitivity C-reactive protein [12]. We suggest that circulating APE1/Ref-1 may have anti-inflammatory effects as in previous other reports [25], and would protect cardiac function against myocardial damage.

In myocarditis, serum biomarkers of inflammation such as leukocytes, erythrocyte sedimentation rate and C-reactive protein are often elevated [7,8]. However, they can’t confirm the diagnosis of myocarditis and normal values in them don’t rule out the diagnosis. Cardiac enzymes also are increased in some patients in myocarditis, but they are non-specific. Cardiac troponins are elevated more commonly [8] and more sensitive about cardiomyocyte injury [2,7,26] but, they are mainly increased in acute early-onset myocarditis, not in long-term myocarditis [2,5]. This also applies to high-sensitivity troponin [5]. NT-proBNP can be elevated in myocarditis. Elevated levels are helpful when heart failure is developed; this is sensitive for heart failure but, it is restrictive for diagnosis and prognosis prediction of myocarditis [5,27]. The utility of viral serology for myocarditis is also limited [2,7]. Coxsackievirus replication is highest after 3 days of infection and decreases soon. In the previous reports, myocardial damage and pre-inflammatory cytokines are dramatically increased in strong association with increasing virus titers at day 3 after infection. Whereas the IL-1b, IFN-b and IL-6 expression was not observed after the virus titer declined at day 14 after infection [28,29,30]. So, it is very difficult to diagnose viral myocarditis in the human patient without the endo-myocardium biopsy. APE1/Ref-1 is correlated with heart virus titers at the late time point without detecting viable viruses. In these respects, elevation of serum APE1/Ref-1 can be helpful for diagnosis of myocarditis, especially at later time. Also, it seems to be able to reflect myocardial injury showing a strong correlation with myocardial inflammation. It can aid to stratify the degrees of myocardial injury without EMB.

Our study has several limitations. Although we measure serum APE1/Ref-1 at several time points, they were not continuously measured values. We couldn’t be sure the peak levels of troponin I, NT-proBNP and APE1/Ref-1. Also, we did not identify the normalization of APE1/Ref-1. We examined it for just 10 days after viral infection. We just focused on the early time point in this time. In the other previous studies, myocardial inflammation with inflammatory cytokines was decreased within 10 or 14 days after infection, suggesting that a period of 10 days after infection is enough to cause significant inflammation [31,32]. Next, there are many various causes that result in acute myocarditis. Our results might be limited to CVB3 induced viral myocarditis. But, CVB3 is a major cause of viral myocarditis.

In conclusion, this study demonstrates the serum APE1/Ref-1 was increased in viral myocarditis till later time. Elevated levels of serum APE1/Ref-1 were strongly associated with the degrees of myocardial inflammation and injury. Our study suggests that serum APE1/Ref-1 can be used to aid in the assessment of diagnosis and myocardial injury in acute viral myocarditis. Further research is needed to verify these results in patients with myocarditis and to evaluate an association with clinical outcome as a prognostic marker.

## 4. Materials and Methods

### 4.1. Ethics Statement

All protocols were reviewed and approved by the Committee on the Ethics of Animal Experiments of Chungnam National University Graduate School of Medicine (CNUH-015-A0023, 1 January 2017). The mice which were used in experiments were given a standard laboratory diet and cared for in accordance with a protocol approved. All efforts were made to minimize their suffering.

### 4.2. Viral Myocarditis Model

Coxsackievirus B3 (CVB3) was derived from the infectious cDNA copy of the cardiotropic H3 strain (CVB3-H3). The five-weeks-old male BALB/c mice (about 20 g in weight, Daehan Biolink, Eumseong, Korea) were infected intraperitoneally with 10^4^ plaque-forming units (PFU) of CVB3-H3 [31]. Blood and hearts were collected on 3 (*n* = 10), 7 (*n* = 10) and 10 days (*n* = 7) after the infection. To compare groups, mice without infection were used as a control group (*n* = 5).

### 4.3. Enzyme-Linked Immunosorbent Assay (ELISA) for the Serum Levels of Troponin I, NT-proBNP and APE1/Ref-1

All blood samples that were taken were centrifuged by 3000 r/min for 10 min at room temperature for serum collection and subsequently stored at −80 °C. The serum was analyzed by the sandwich ELISA kit for Troponin I (Kamiya biomedical company, Tukwila, WA, USA), NT-proBNP (Elabscience, Wuhan, China) and APE1/Ref-1 (LifeSpan BioSciences, Inc., Seattle, WA, USA) following the manufacturer’s instruction. 

### 4.4. Organ Virus Titers

We homogenized the hearts in Dulbecco’s Modified Eagle’s Medium (DMEM) containing 4% fetal calf serum (FCS). The cellular debris was removed by centrifugation at 300× *g* for 10 min. The viral titers in the supernatants were determined by PFU assay using HeLa cells. 

### 4.5. Histopathology

The hearts were fixed in 10% formalin, embedded in paraffin and stained with hematoxylin and eosin (H & E). Four researchers, blinded to the study, graded the prepared sections for myocardial inflammation as follows: grade 0 represented no myocarditis, 1 represented 1 to 10 lesions of focal myocardial inflammation in high power fields (×400), 2 represented 11 to 20 lesions, 3 represented 21 to 40 lesions, and 4 represented widespread and confluent inflammation [33].

### 4.6. Statistical Analysis

The data were analyzed using standard software (SPSS version 20.0, IBM Co., Chicago, IL, USA). All data were presented as mean ± standard error of the mean (SEM). Statistical evaluation was conducted using a one-way analysis of variance followed by Dunnett post-hoc test. Pearson’s correlation analyses were performed to determine the association among variables. A *p* value less than 0.05 was considered statistically significant.

## Abbreviations

APE1/Ref-1	Apurinic/apyrimidinic endonuclease 1/redox effector factor-1
NT-proBNP	*N*-terminal pro-B-type natriuretic peptide
EMB	Endomyocardial biopsy
CVB3	Coxsackievirus B3
PFU	Plaque-forming units
ELISA	Enzyme-Linked Immunosorbent Assay
